# The Influence of Sweet Taste Perception on Dietary Intake in Relation to Dental Caries and BMI in Saudi Arabian Schoolchildren

**DOI:** 10.1155/2017/4262053

**Published:** 2017-08-21

**Authors:** Heba Ashi, Guglielmo Campus, Heléne Bertéus Forslund, Waleed Hafiz, Neveen Ahmed, Peter Lingström

**Affiliations:** ^1^Department of Cariology, Institute of Odontology, Sahlgrenska Academy, University of Gothenburg, Gothenburg, Sweden; ^2^Department of Preventive Dentistry, Faculty of Dentistry, King Abdulaziz University, Jeddah, Saudi Arabia; ^3^Department of Surgery, Microsurgery and Medicine, School of Dentistry, University of Sassari, Sassari, Italy; ^4^WHO Collaborating Center for Epidemiology and Preventive Dentistry, Milan, Italy; ^5^Department of Internal Medicine and Clinical Nutrition, Institute of Medicine, Sahlgrenska Academy, University of Gothenburg, Gothenburg, Sweden; ^6^Department of Prosthodontics, North Jeddah Speciality Dental Center, Jeddah, Saudi Arabia; ^7^Department of Pedodontics, Jeddah Speciality Dental Center, Jeddah, Saudi Arabia

## Abstract

**Objectives:**

The aim of the study was to evaluate the influence of sweet taste perception on dietary habits in Saudi schoolchildren. In addition, the relationship between dietary habits and both caries and BMI was studied.

**Methods:**

A cross-sectional observational study comprising 225 schoolchildren aged 13–15 years from Jeddah, Saudi Arabia, was conducted. The consumption frequency of certain food items was analysed from a beverage and snack questionnaire and a three-day estimated dietary record was obtained. The sweet taste perception level was determined as sweet taste threshold (TT) and sweet taste preference (TP). Children were grouped into low, medium, and high, according to their sweet taste perception level. ICDAS and DMFS indices were used for caries registration and anthropometric measurements using BMI were collected.

**Results:**

Sweet taste perception was found to be negatively correlated to the number of main meals and positively correlated to both snack and sweet intake occasions. Statistically significant differences were found between the TT and TP groups with regard to the number of main meals and sweet intake (*p* ≤ 0.01). No significant correlation between the dietary variables and caries or BMI was found.

**Conclusions:**

The dietary habits and sweet intake were found to be influenced by the sweet taste perception level, while the relation between the dietary habits and the caries and BMI was found insignificant.

## 1. Introduction

A healthy diet is of significant importance for growth, development, and prevention from dietary-related diseases. There are different medical and oral conditions that are known to be related to dietary imbalance. These conditions include dental caries and obesity which are in turn known to affect general health [1–5].

Dietary habits and food intake have been found to be influenced by several factors. These factors include taste perception, which in turn can be affected by cultural and genetic factors [6–10]. In addition, the frequent consumption of a certain food type can change this perception to be habituated to the taste and increases the preference for this food item [11–13].

One of the main dietary items known for its unfavourable effect is sugar, and that when it is consumed in amounts above the recommended levels. A diet rich in sugar can affect the teeth at an early age and continues towards adolescence causing several oral problems such as dental caries [4, 5]. Both the amount and frequency of sugar consumption may have an adverse effect on the teeth [5, 14–16]. In addition, dietary habits such as frequent snacking have been found to be related to overweight, due to the high content of fat and sugars in snacks [1, 17]. Several studies have suggested that sugar intake is determined by several factors such as environmental and genetic factors [18–21].

Unhealthy dietary habits are common worldwide and especially in children. It has been found in many cultures that children do not follow the recommendations with regard to diet, and sometimes the dietary habits are at an acceptable level but still does not reach the optimum [22–24]. Regarding Saudi Arabia, poor dietary choices among schoolchildren have been reported, with regular sweet food and beverage consumption [25–27]. This can be attributed to the national increase in globalisation, westernisation, and economic and social revolution [28, 29].

The aim of this study was to evaluate the influence of sweet taste perception on dietary habits in Saudi schoolchildren aged 13–15 years. In addition, the relationship between dietary intake and caries, as well as BMI, was studied. Our aim was based on the hypothesis that sweet taste perception can affect children's dietary habits, which may in turn have an effect on both oral and general health.

## 2. Materials and Methods

### 2.1. Subjects and Study Design

The study was a cross-sectional observational investigation. It is a part of a broad multicenter study conducted in Italy, Mexico, and Saudi Arabia aimed to investigate the different taste perception level, dental caries, and BMI. Children from Jeddah, Saudi Arabia, who were included in the sample selected in the previous studies (33.6%) were further assessed regarding their dietary habits. This study included 225 children (114 boys/111 girls), aged 13–15 years, who were randomly selected from school lists entered into a computerized program. The boys and girls were selected from separate school lists as they were attending different schools. Children living in the country from six years of age were included in the survey. However, children with medical conditions or undergoing orthodontic treatment were excluded. In addition, any child with signs of flu at the time of the study was excluded, as flu can affect taste sense. The students were chosen using a computerized program and 1% of the population inserted into the program was randomly selected. A post hoc power analysis with a noncentrality parameter of 18, a critical *χ*^2^ of 7.81, and a power (1-*β* err prob) of 0.96 was performed.

Ethical approval was obtained from the school of dentistry ethical committee at King Abdulaziz University (029-12). In addition, school entry and data collection permission was given by the Board of Education in Jeddah, Saudi Arabia. Parent's consent forms were signed prior to the start of the study and children were informed about the process of the data collection. Data collection was conducted in school settings and all the students were examined in their classrooms under natural light.

### 2.2. Estimated Food Record

Dietary information was collected via a three-day food and beverage diary presenting daily variations in intake. Participants were asked to report any food or drink consumed in terms of time and quantity from two weekdays and a day from the weekend. When possible, the event, whether it was regarded as breakfast, lunch, or dinner, was stated by the children.

The documents were handed to the participants at school and collected and reviewed by the main author and coauthor. The numbers of main meals (structured eating event), snacks (unstructured eating event and usually uncooked), and total intake occasions (sum of main meals and snacks intakes) were then retrieved and calculated from the dietary record. Any food and beverages consumed within 30 minutes were regarded as one intake event [30]. Subsequently, each event was classified as either a main meal or a snack, depending on the time and type of consumption. In addition, sweet intake frequency was assessed separately by counting all food and drinks containing sweet substances reported in the diary. No total energy intake evaluation was made.

### 2.3. Beverage and Snack Questionnaire

The food frequency questionnaire was adapted from the validated self-administered beverage and snack questionnaire [31, 32]. A total of 19 food and drink items (sugar-sweetened beverages, savoury snacks, and sweets) were included in the questionnaire. For data analysis, nine sweet-containing beverages and snacks were included (five beverages and four snacks). Students were asked to choose from seven options ranging from never (zero intake) to more than four times per day. Afterwards, a score was given to each option presented in the beverage and snack questionnaire (i.e., never or zero intake = 0, once a week = 1). The scores for the nine sweet items were then added for each participant and summed as a beverage sweet intake score (BS) and a snack sweet intake score (SS).

### 2.4. Sweet Taste Perception Test

The sweet taste perception level of the participant was determined using a modified version of Furquim et al. [33] method, originally described by both Nilsson and Holm [34] and Zengo and Mandel [35]. Two variables were to be described in relation to sweet taste perception; the first is the sweet taste threshold, defined as the first concentration at which the participants can identify the presence of sucrose and distinguish it from water and the second was the sweet taste preference, which is their preferred chosen solution that matches the level of sugar they might like in a drink. The participants tasted all ten different sucrose solutions with concentrations ranging from 1.62 to 821.52 gm/L. Between tasting the children rinsed with filtered water.

The solutions were tested in order of increasing concentration, after which children were asked to identify their sweet taste threshold level and sweet taste preference of the same solutions and mark the respective solution number on a sheet they were given. Instructions were given to use 10 ml of the offered solution, to guarantee exposing all the taste buds in the oral cavity for at least five seconds, and then to spit it out.

The children were subsequently grouped depending on the concentration chosen for the sweet taste preference (TP) and sweet taste threshold (TT) test, respectively. The children were regarded as “low,” when they chose a solution in the range of 1.63–12.84 gm/L, “medium,” when the chosen solution ranged from 25.67 to 102.69 gm/L, and “high,” when the solution with a concentration of 205.38–821.52 gm/L was chosen.

### 2.5. Caries Registration

The number of decayed, missed, and filled tooth surfaces (DMFS) and the International Caries Detection and Assessment System (ICDAS) indices were used to diagnose caries. The DMFS index is defined as the sum and score of the total decayed, missed, and filled tooth surfaces of the subjects.

Regarding the ICDAS index, it constitutes scoring system of two digits (restoration code and tooth status). The second digit is the code for the tooth surface status scored from 1 to 6 with 1 presented as first visual change in enamel and 6 is distinctive lesion into dentine. The visible tooth change in enamel due to caries was defined as initial caries (ICDAS 1, 2, and 3) and any lesion reaching the dentine was manifest (ICDAS 4, 5, and 6).

All the children were examined in the classroom by the main author (HA) or one of the coauthors (WH). Disposable dental examination kits, gloves, and masks were used, according to WHO criteria. No radiographs were taken due to the school-setting nature of the study and examinations were performed under adequate natural light.

### 2.6. Anthropometric Measurements

Data on height (cm) and weight (kg) were obtained and body mass index (BMI; WHO BMI-for-age) [36–38] was evaluated for each study participant. Subsequently, BMI was calculated as body weight divided by height squared. Portable scales were used and the children were instructed to remove all shoes, socks, and heavy clothing when measured.

### 2.7. Statistical Analysis

The mean, standard deviation, and range for the variables were analysed using IMB® SPSS® (PASW version 23.0 IBM® Chicago, Ill, USA). The independent* t*-test was used to determine the differences between boys and girls. Differences between the sweet taste perception groups (low, medium, and high) were tested by one-way ANOVA. Regarding beverage and snack questionnaire, the BS and SS scores were considered as a dependent variable and used for multiple linear regression analysis. The relationship between variables was tested using Spearman's rank correlation. A *p* value of <0.05 was considered statistically significant.

## 3. Results

From the dietary record, it was found that the number of main meal events constitutes the largest part of the total daily intake events among Saudi children, followed by the snack intake events (Table 1). The majority of all eating and drinking occasions included the consumption of some sweet items (Table 1).

### 3.1. Sweet Taste Perception and Dietary Habits

An analysis of the correlation between sweet taste perception and dietary intake showed a significant yet weak to moderate negative correlation between the number of main meals and the sweet taste threshold and sweet taste preference, respectively (*p* ≤ 0.001; *r* = −0.228 and *p* ≤ 0.001; *r* = −0.480). In terms of the snack occasions and sweet intake occasions, a significant weak positive correlation was found for the sweet taste threshold (*p* ≤ 0.05; *r* = 0.169 and *p* ≤ 0.001; *r* = 0.276) and sweet taste preference, respectively (*p* ≤ 0.001; *r* = 0.286 and *p* ≤ 0.001; *r* = 0.288). No correlation was found between any of the three intake variables (number of main meals, snack events, and sweet intake events) and both caries and BMI, respectively.

**Figure 1:**
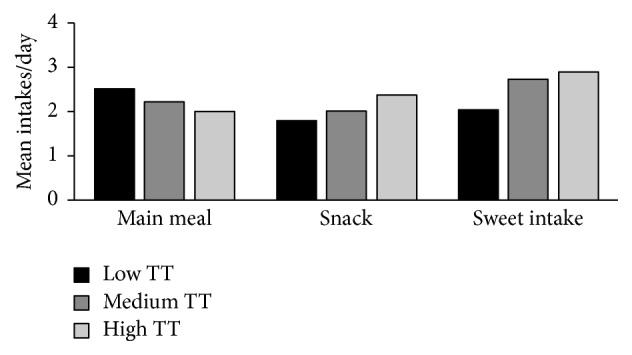
Mean value for main meal, snack, and sweet intake in sweet taste threshold groups. *n* = 225.

In terms of the sweet taste threshold groups, the high sweet taste threshold group had the highest sweet intake and number of snack occasions and the lowest main meal frequency (Figure 1, Table 2). A statistically significant difference between the three groups was found for the number of main meals (*p* ≤ 0.01) and sweet intake occasions (*p* ≤ 0.001), (Table 2).

Similarly, regarding the grouping of children according to their sweet taste preference, statistically significant differences were found between the groups with regard to the number of main meals (*p* ≤ 0.001) and sweet intake occasions (*p* ≤ 0.001) (Table 3). In addition, the high sweet taste preference group was found to have a higher number of snacking and sweet intake occasions and the lowest number of main meals (Table 3).

### 3.2. Beverage and Snack Questionnaire

Fruit juice consumption showed a weak significant negative correlation to sweet taste threshold (*p* ≤ 0.05; *r* = −0.160) and sweet taste preference, respectively (*p* ≤ 0.01; *r* = −0.232) for all children. In addition, sports drink intake was moderately correlated to both sweet taste threshold and sweet taste preference (*p* ≤ 0.01; *r* = 0.295; *p* ≤ 0.01; *r* = 0.342). Regular soda showed weak positive correlation to sweet taste preference (*p* ≤ 0.05; *r* = 0.178). Regarding snacks and sweet taste perception, sweet chocolates and gummies sweets showed a significant weak positive correlation to the sweet taste threshold (*p* ≤ 0.05; *r* = 0.151) in all subjects.

Children who have a higher weekly intake of fresh juice had a lower BMI (*p* ≤ 0.05; *r* = −0.135) and those with a higher intake of ice cream had a lower DMFS (*p* ≤ 0.05; *r* = −0.139). None of the other food or drink items from the questionnaire showed any significant correlation with the dietary variables, BMI, or caries.

The BS was significantly associated with gender and sweet intake occasions (Table 4). Regarding SS, no significant association was found (data not shown). However, gender showed a borderline significance (*p* = 0.059, 95% CI = −0.044–2.34).

### 3.3. Gender Differences

A statistically significant difference was found between boys and girls for the number of main meals and snacking events (*p* ≤ 0.01, resp., *p* ≤ 0.001). The number of main meals was higher in girls, while the snacking occasions were found to be higher among boys (Table 1). However, boys reported a higher total number of intake occasions compared with girls (Table 1). Regarding sweet intake occasions, a higher intake was found among the boys (*p* ≤ 0.001) (Table 1).

Statistically significant differences were found between boys and girls for the sweet taste threshold (*p* ≤ 0.001), sweet taste preference (*p* ≤ 0.001), and BMI (*p* ≤ 0.01), with a higher mean value among boys (Table 5). Regarding the caries variables, girls had a higher mean value for initial lesions compared with boys (*p* ≤ 0.001), while higher mean values for DMFS and manifest lesions were found among boys (Table 5).

## 4. Discussion

In this study, the influence of sweet taste perception on dietary habits was evaluated as part of a multicenter study focusing on sweet taste perception in relation to different tested variables. The main hypothesis was supported by our findings, as the sweet taste threshold and preference were found to be directly related to both snack and sweet intake occasions and inversely related to the number of main meals. This is supported by Kourouniotis et al. [13], where the taste perception was found to affect diet and that subjects considering taste as an important factor in their food choices tend to consume more sweets and snacks with a high fat and sugar content.

Moreover, when children were categorised according to their sweet taste perception level, the group with the higher threshold and preference for sweets reported a higher number of sweet and snack intake occasions and a lower number of main meals. This could be due to the higher threshold for sweets over time, which leads to increased consumption in order to reach the preferred sweet taste and corresponds well to what was previously stated, “taste perception can result from a cumulative effect of lifetime exposure to certain food or taste” [13].

Children usually run a higher risk of adaptation to unhealthy dietary behaviours with an increase in sugar intake. Childhood is known to be a period with a high preference for sweets due to several factors, which may lead to food choices rich in calories [2, 20, 21, 39]. However, this sweet preference has been found to change with age, as less energy is needed [21].

The second hypothesis of the present study focused on the relationship between dietary patterns and caries. No significant relationship was found in this study between sweet intake occasions from the dietary record and any of the caries variables, despite the well-known relationship between sugar intake and caries [5, 14–16]. A possible explanation could be attributed to the cross-sectional design, where children's dietary assessment was conducted on a period of one week and thus reflects the present dietary habits with no adequate information of the past diet history. Meanwhile, caries is the net result of a longitudinal metabolic process that requires several factors contributing over time. Thus, the possible previous dietary factors affecting the children's caries level are not known in this study. However, in a previous study by our group, including the same children from Saudi Arabia, a relationship was found between sweet taste perception and DMFS.

Regarding the relationship between dietary patterns and BMI, no significant relationship was found in this study. However, a study by Washi and Ageib [17] stated that a significant relationship was reported between the number of meals and being overweight. Despite the fact that no relation was found in this study between the dietary habits and BMI or caries, it is well known from literature that both diseases can be affected by the dietary intake [1, 5, 14–17].

An association has been found in this study between the beverage sweet intake score (BS) and both the gender and sweet intake occasions and it can be attributed to the difference in behaviour presented in the population with regard to gender. This can also be seen when calculating the mean number of sweet intake occasions from the dietary records, where males reported a higher number of sweet intake occasions. Our findings may imply that the same dissimilarity can be seen between boys and girls in both the questionnaire and the food diary. Unhealthy habits in Saudi children have previously been discussed in the literature [25–27]. Due to the lifestyle and the cultural and environmental aspects in Saudi Arabia, the risk of an unhealthy lifestyle has previously been found especially high among females [27, 40–43]. In Saudi, fewer chances are offered for exercise, especially to females, which may cause an increased risk of obesity, as well as unhealthy dietary habits [43].

Moreover, differences between genders have been reported with regard to sweet taste perception. Females considered taste to be of great importance in food choices compared with males and this was linked to poor dietary habits [13, 43]. Conversely, our study revealed a difference in dietary pattern between boys and girls, with more positive habits among girls. Boys had a higher number of sweet and snack intake occasions and this is contrary to the findings in a study performed in Kuwait by Allafi et al. [44] where no differences between boys and girls were found with regard to sugar consumption. The differences found between boys and girls in the present study may be related to the higher reported sweet taste threshold and preference. In addition, higher BMI and DMFS values were found among the boys and this might also be related to the reported liking for sweets.

Regarding the beverage and snack questionnaire, the items with a sweet taste were included in the analysis, as this was the focus of our study. Children with a higher sweet taste threshold had a higher consumption of chocolates, gummy candies, and sports drink. A similar finding was made by Kourouniotis et al. [13], where more chocolates and sweets were consumed by those reporting taste as important. This could be explained by poor dietary choices being influenced by the high sweet taste threshold, so that more calories are consumed from sources other than proper cooked meals.

The children's perception of the importance of a healthy diet and why it should be adapted was not evaluated in this study, but it is of interest for further studies. It has previously been reported that only 11.3% of the children were concerned about general health, while the majority were concerned about looks and obesity [27].

The majority of children followed the instructions, but personal variations in presentation and reporting cannot be totally excluded. Even if the days reported in the dietary record were representative, the possibility cannot be excluded that certain food intakes were not identified. It is difficult to obtain an exact count of frequency in dietary records and especially at this age. Regarding the beverage and snack questionnaire, the exposure of a food item can be interpreted in different ways by different subjects. Items that were not listed in the questionnaire may have been consumed, as there are a limited number of items. In addition, limitations apply to any self-administered questionnaire and data should be handled with caution.

In a step towards a healthier lifestyle, Saudi Arabia banned carbonated drinks from school, but this did not apply to all sweetened beverages [28], as these drinks have been found to be linked to poor dietary choices [26]. In addition, special attention should be paid to children and adolescents, as this is the period in life where rapid growth, maturity, and lifelong habits may develop [17, 44].

## 5. Conclusions

Dietary habits and especially sweet intake in Saudi children were influenced by the sweet taste perception level, which may in turn have an adverse effect on health. However, no relation was found between the dietary habit and caries or BMI. Therefore, further longitudinal study design is recommended in this regard.

In addition, boys had poor dietary habits in comparison with girls, as well as a higher caries prevalence and BMI. This emphasises the effect of taste sense, as boys showed higher sweet taste perception than girls.

A healthier diet with a low sugar content is therefore of great importance for improved oral and general health. The recommendations are to try and control the food served at schools and public-health attention is needed to target children and adolescents to encourage them to adapt proper dietary habits and a healthier lifestyle. Further studies focusing on an analysis of actual energy intake in the diet are needed.

## Figures and Tables

**Table 1:** Mean and standard deviation (SD) for main meal, snack, total intake, and sweet intake in Saudi schoolchildren (boys, girls).

Variable	Boys (*n* = 114)		Girls (*n* = 111)		All (*n* = 225)		*p* value^(1)^
	Mean ± SD	Range	Mean ± SD	Range	Mean ± SD	Range	
Main meal	2.2 ± 0.7	1–3	2.5 ± 0.6	1–3	2.3 ± 0.7	1–3	0.003
Snack	2.2 ± 0.8	0–4	1.7 ± 0.7	0–3	1.9 ± 0.8	0–4	0.000
Total intake	4.4 ± 0.9	2–7	4.1 ± 0.8	3–6	4.2 ± 0.9	2–7	0.046
Sweet intake	2.8 ± 1.1	0–6	2.1 ± 1.4	0–5	2.4 ± 1.3	0–6	0.000

^(1)^ *p* value: significance between boys and girls (independent *t*-test).

**Table 2:** Mean and standard deviation (SD) for main meal, snack, total intake, and sweet intake in Saudi schoolchildren according to TT (taste threshold) groups (low, medium, and high).

Variable	Low TT (*n* = 93)		Medium TT (*n* = 124)		High TT (*n* = 8)		*p* value^(1)^
	Mean ± SD	Range	Mean ± SD	Range	Mean ± SD	Range	
Main meal	2.5 ± 0.6	1–3	2.2 ± 0.7	1–3	2.0 ± 0.5	1–3	0.002
Snack	1.8 ± 0.7	1–3	2.0 ± 0.9	0–4	2.4 ± 0.9	1–3	0.063
Total intake	4.3 ± 0.8	3–6	4.2 ± 1.0	2–7	4.4 ± 1.2	3–6	0.675
Sweet intake	2.0 ± 1.3	0–5	2.7 ± 1.2	0–6	2.9 ± 1.6	1–5	<0.001

^(1)^ *p* value: significance between TT groups (ANOVA).

**Table 3:** Mean and standard deviation (SD) for main meal, snack, total intake, and sweet intake in Saudi schoolchildren according to TP (taste preference ) groups (low, medium, and high).

Variable	Low TP (*n* = 11)		Medium TP (*n* = 89)		High TP (*n* = 125)		*p* value^(1)^
	Mean ± SD	Range	Mean ± SD	Range	Mean ± SD	Range	
Main meal	3.0 ± 0.0	1–3	2.7 ± 0.5	2-3	2.0 ± 0.7	1–3	<0.001
Snack	1.8 ± 1.0	0–3	1.8 ± 0.6	1–3	2.1 ± 0.9	0–4	0.026
Total intake	4.8 ± 1.0	3–6	4.4 ± 0.7	3–6	4.1 ± 1.0	2–7	0.003
Sweet intake	2.4 ± 0.7	2–4	2.0 ± 1.3	0–5	2.8 ± 1.2	0–6	<0.001

^(1)^ *p* value: significance between TP groups (ANOVA).

**Table 4:** Multiple linear regression analysis for factors associated with the beverage sweet intake score (BS).

Variables	*B*	Std. error	*t*	Sig.
TT	.001	.003	.434	.665
TP	.001	.001	1.402	.162
Sweet intake	.322	.093	3.470	.001
Snack	.116	.183	.638	.524
Main meals	−.322	.229	−1.406	.161
Gender	.813	.308	2.637	.009

**Table 5:** Mean and standard deviation (SD) for sweet taste threshold (TT), sweet taste preference (TP), BMI, initial, manifest lesions, and DMFS in Saudi schoolchildren (boys, girls).

Variable	Boys (*n* = 114)		Girls (*n* = 111)		All (*n* = 225)		*p* value^(1)^
	Mean ± SD	Range	Mean ± SD	Range	Mean ± SD	Range	
TT (gm/L)	52.1 ± 58.7	1.6–410.8	22.9 ± 24.9	1.6–205.4	37.7 ± 47.5	1.6–410.8	0.000
TP (gm/L)	431.3 ± 316.3	1.6–821.5	205.1 ± 237.5	6.5–821.5	319.7 ± 302.1	1.6–821.5	0.000
BMI	25.1 ± 6.9	14.7–44.5	22.6 ± 4.8	12.6–37.7	23.9 ± 6.1	12.6 ± 44.5	0.002

Initial lesions	4.9 ± 4.6	0–21	8.6 ± 9.8	0–59	6.7–7.8	0–59	0.000
Manifest lesions	1.7 ± 3.6	0–24	0.9 ± 1.6	0–9	1.3 ± 2.8	0–24	0.025
DMFS	3.1 ± 4.5	0–25	2.9 ± 3.5	0–22	3.0 ± 4.0	0–25	0.679

^(1)^ *p* values: significance between boys and girls (independent *t-*test).
